# Direct Writing of Cu Patterns on Polydimethylsiloxane Substrates Using Femtosecond Laser Pulse-Induced Reduction of Glyoxylic Acid Copper Complex

**DOI:** 10.3390/mi12050493

**Published:** 2021-04-27

**Authors:** Nam Phuong Ha, Tomoji Ohishi, Mizue Mizoshiri

**Affiliations:** 1 Department of Mechanical Engineering, Nagaoka University of Technology, Nagaoka, Niigata 940-2188, Japan; s183068@stn.nagaokaut.ac.jp; 2 Shibaura Institute of Technology, Shibaura Institute of Technology, Tokyo 135-8548, Japan; ooishi@mte.biglobe.ne.jp

**Keywords:** femtosecond laser pulse-induced reduction, copper wire, glyoxylic acid copper complex, polydimethylsiloxane

## Abstract

We investigate the direct writing properties of copper (Cu) patterns on glass and polydimethylsiloxane (PDMS) substrates using femtosecond laser pulse-induced thermochemical reduction of glyoxylic acid copper (GACu) complex. The films of the GACu complex coated on the substrates were irradiated by focused femtosecond laser pulses using a low numerical aperture of 0.45. Under the same conditions, such as laser scanning speed and pulse energy, the width of the line patterns fabricated on PDMS substrates was larger than that on glass substrates. X-ray diffraction peaks of the patterns on glass substrates corresponded to Cu without significant oxidation. By contrast, although Cu patterns were fabricated on PDMS substrates at a scanning speed of 10 mm/s and pulse energy of 0.49 nJ, Cu_2_O was also generated under overheating conditions at a scanning speed of 1 mm/s and pulse energy of 0.37 nJ. All the patterns exhibited electrical conductivity. The minimum resistivity of the patterns on PDMS substrates is 1.4 × 10^−5^ Ωm, which is 10 times higher than that on glass substrates, indicating that microcracks formed by thermal shrinkage of the substrates during the laser irradiation increase the resistivity. This direct Cu writing technique on soft materials is useful for fabricating flexible microdevices.

## 1. Introduction

Printing technology on flexible substrates has received attention for manufacturing wearable devices. Laser direct writing technique is a promising method for fabricating metal patterns in air because formation and metallization are simultaneously performed by laser irradiation. To date, various inks—such as metal and metal oxide nanoparticle (NP) inks—and lasers are used for laser direct writing of metals in air. Metal NP inks—such as Ag- and Cu-NP inks—have been sintered to form arbitral patterns by scanning focused laser light [1,2,3]. In addition, metal oxide NP inks—such as CuO- and NiO-NP inks—which are more stable in air, have also been developed for laser direct writing [4,5,6]. For example, CuO-NP inks—comprising CuO-NPs, ethylene glycol (EG) as a reductant, and polyvinylpyrrolidone (PVP) as a dispersant—were spin-coated to form films [4]. Then, continuous-wave or nanosecond laser lights were focused and scanned onto the films to form Cu patterns using a thermochemical reduction. Ni patterns are also fabricated in air using reductive sintering of NiO-NPs. NiO-NP inks were prepared by mixing NiO-NPs and toluene as a reductant [5,6]. Cu_2_O-NP as a raw material and other combinations of reductants and dispersants—such as 2-propanol and PVP—have also been used in the laser direct writing technique [7].

Metal complex inks are developed for laser direct writing [8,9,10]. An advantage of using metal complex inks is that the metal patterns are fabricated without significant oxidation. Glyoxylic acid copper (GACu) complex is a promising candidate because the only inert gas—such as CO_x_ and H_2_O—are generated in its reaction processes [9,10]. However, the patterns are formed on glass substrates because CO_2_ lasers used to induce thermochemical reduction for Cu precipitation are expected to damage soft substrates.

Moreover, we developed femtosecond laser reductive sintering of metal oxide NPs to write metal patterns on glass substrates in air. The process allowed us to control the degree of reduction of metal oxide NPs. For example, CuO-NPs in the inks were selectively reduced and sintered to Cu-rich and Cu_2_O-rich patterns in an ambient atmosphere by controlling the laser irradiation conditions, such as laser scanning speed and pulse energy [11,12]. The resistivities of the Cu-rich and Cu_2_O-rich patterns exhibited metal- and semiconductor-like properties, which increased and decreased with the increase in temperature, respectively [11]. The controlling of the reduction and reoxidation of CuO/NiO mixed NPs enabled us to fabricate Cu_2_O/NiO-rich and Cu-Ni-rich patterns that showed p- and n-type thermoelectric properties, respectively [13]. Further, Cu patterns were fabricated on glass substrates using femtosecond laser pulse-induced reduction of GACu complex [14]. When the femtosecond laser pulses were focused onto the GACu complex films coated on glass substrates using an objective lens with a high numerical aperture (NA) of 0.80, the multiphoton absorption caused only around the focal spot. The Cu precipitation mainly caused by the multiphoton absorption-induced thermochemical reduction because the precipitated area was larger than the laser-irradiated one. The focal spot diameter is estimated by the diffraction limit, 1.22*λ*/NA (*λ*: wavelength). Therefore, the high resolution achieves using a high NA objective lens than using a low NA objective lens. However, the short focal depth (twice the Rayleigh length) of the laser pulses using the high NA objective lens is restricted to fabricating Cu patterns on the hard substrates with small linear thermal expansion and on planar surfaces on which the laser pulses can be focused precisely.

In this study, we investigated the properties of Cu patterns fabricated by femtosecond laser pulses focused on glass and polydimethylsiloxane (PDMS) substrates using an objective lens with a low NA of 0.45. The morphology, line width, crystal structures, and resistivity of the patterns fabricated on glass and PDMS substrates were evaluated. Finally, a Cu pattern was demonstrated on a PDMS step structure.

## 2. Materials and Methods

### 2.1. Femtosecond Laser Direct Writing of Cu Wires

**Figure 1:**
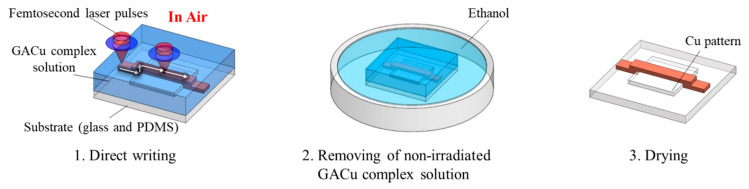
Schematic illustration of the femtosecond laser direct writing on glass or PDMS substrates.

Figure 1 illustrates the femtosecond laser direct writing process on glass and PDMS substrates. First, GACu complex solution was prepared using a previously reported method [9,10,14]. Glyoxylic acid (GA) solution consisting of 4.5-mmol GA and 5-mL H_2_O was adjusted to pH 7.0 by adding NaOH aqueous solution. Then, 4.5-mmol Copper (II) sulfate pentahydrate [CuSO_4_·5H_2_O] dissolved in 5-mL H_2_O was added to the GA solution and stirred for 3 h. The precipitation of GACu complex was filtered and washed with H_2_O using centrifugal separation. Next, the GACu complex was dried using a vacuum freeze drier (VFD-03, AS ONE Corporation, Osaka, Japan). Afterward, 6 mmol of the dried GACu complex was dissolved in 2-mL ethanol and 1-mL 2-aminoethanol mixed solution. 

Next, the GACu complex solution was spin-coated on glass and PDMS substrates. The PDMS substrates were prepared by curing liquid PDMS prepolymer mixed with a curing agent with a 10:1 concentration ratio. PDMS substrates with a thickness of 2 mm were formed by cross-linking at 150 °C in an oven.

Femtosecond laser pulses operating with a pulse duration, wavelength, and repetition frequency of 120 fs, 780 nm, and 80 MHz, respectively, were focused into the GACu complex films using an objective lens with an NA of 0.45. Such a low NA objective lens is expected to be useful for direct writing on nonplanar surfaces, such as steps, or soft materials with high linear coefficients of expansion because of their long depth of focus. The sample surfaces were observed using a CCD camera which was collimated with the femtosecond laser pulses. By considering the focal shift induced by the difference between the refractive indices of the GACu complex (approximately 1.5) and air (1.0), the laser pulses were focused onto the surface of the substrates using a mechanical stage. The diameter of the focal spot was estimated to be 2.1 µm by considering that the laser pulses with the Gaussian intensity distribution were focused using an objective lens with the NA of 0.45. After irradiating the laser pulses on the substrates, the non-irradiated GACu solution was removed by rinsing in ethanol. Finally, the sample substrates were dried by air blow.

### 2.2. Evaluation of the Patterns

The morphology of the patterns fabricated by irradiating femtosecond laser pulses was evaluated using an optical microscope and a scanning electron microscope (SEM, FlexSEM 1000 II, HITACHI High Technology Corporation, Tokyo, Japan). Crystal structures of the patterns were examined by using a powder X-ray diffraction (XRD) analysis (Rigaku Corporation, MiniFlex) with Cu-Kα radiation. The beam size was approximately 10 mm. The resistivity of the patterns was evaluated by measuring the resistance using a two-probe method (Keysight Technology, Truevolt series 34465) and the heights of the structures were measured using a profilometer (Dektak 6M, ULVAC, Kanagawa, Japan) and a laser probe profilometer (NH-3SP, Mitaka kohki, Tokyo, Japan).

## 3. Results and Discussion

### 3.1. Morphology and Width of Line Patterns and on Glass and PDMS Substrates 

**Figure 2:**
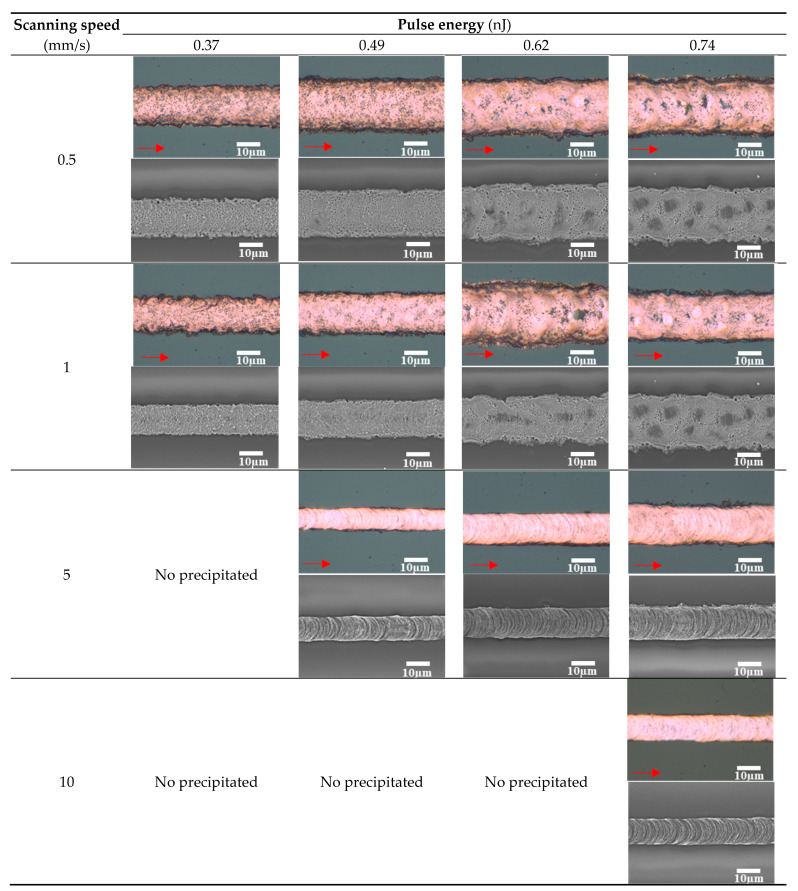
Optical and SEM microscope images on glass substrates at a scanning speed of 0.5–10 mm/s and pulse energy of 0.37–0.74 nJ.

Figure 2 shows the morphologies of the line patterns on glass substrates. A fine line pattern with a width of 10.8 µm was fabricated on glass substrates at a high scanning speed of 5 mm/s and low pulse energy of 0.49 nJ. Remarkable pores were not observed, which is consistent with the results of the previous study using a high NA objective lens of 0.80 [14]. The wavelike features on the surface of the lines seem to be induced by the keyhole formation and melt flow [15]. By contrast, the low dense line patterns with many pores were formed at low scanning speed. These results indicate that overheating of GACu complex solution caused evaporation of the precipitated Cu. The laser irradiation conditions of high scanning speed with high pulse energy are effective to fabricate dense Cu patterns due to flash heating and low heat accumulation.

**Figure 3:**
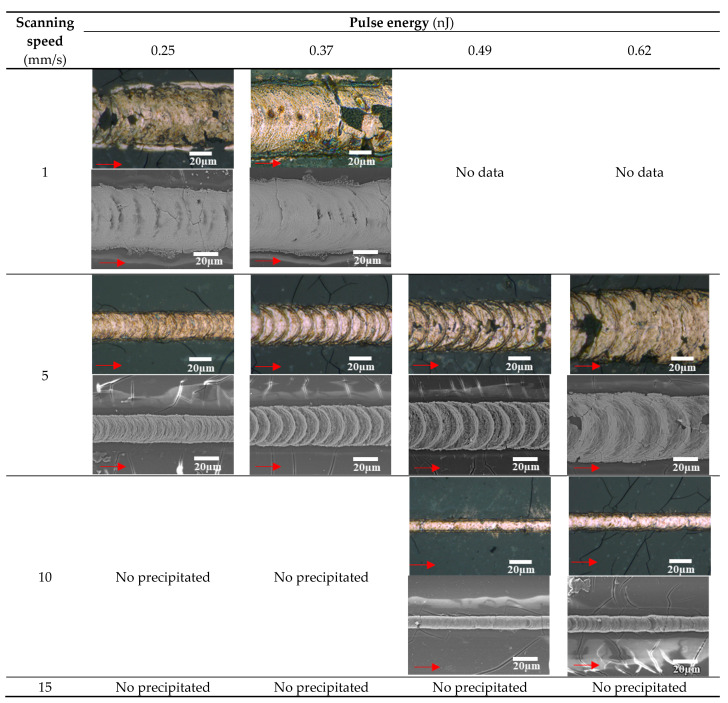
Optical and SEM microscope images on PDMS substrates at a scanning speed of 0.5–10 mm/s and pulse energy of 0.25–0.62 nJ.

Figure 3 shows the optical and SEM images of the line patterns fabricated on PDMS substrates. Continuous line patterns were fabricated on the PDMS substrates without melting at high scanning speed and pulse energy, although some cracks were formed around the lines on the PDMS substrates. The minimum line width is 11.1 µm at a scanning speed of 10 mm/s and pulse energy of 0.49 nJ, which is larger than that on the glass substrates. In addition, the scanning speed on the line width had a significant impact on the line widths. The line patterns seem to be fixed on PDMS substrates by an anchor effect, not melting, by considering the stripped pieces of patterns fabricated at a scanning speed of 1 mm/s. 

When the femtosecond laser pulses were focused onto the substrate surface coated with GACu complex, the multi-photon absorption-induced thermal reduction induced the generation of Cu nanoparticles as the same in Ref. [14]. By considering the direct writing properties, the substrates were not significantly damaged by laser irradiation. Therefore, an anchor effect contributed to the adhesion of Cu to both glass and PDMS substrates.

**Figure 4:**
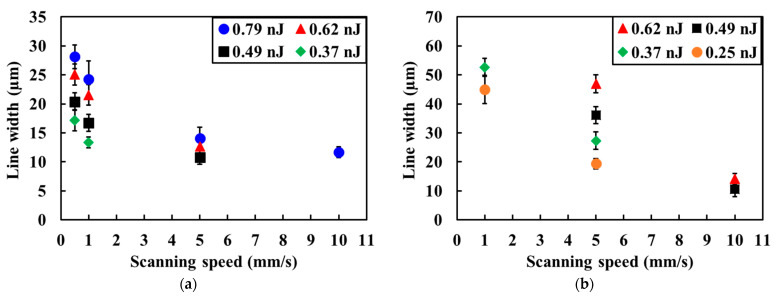
Dependence of scanning speed on line width of the patterns formed at various pulse energies for (**a**) glass substrates and (**b**) PDMS substrates.

The effect of the substrates on the line width was evaluated by measuring the line width from optical microscope images of the line patterns on glass and PDMS substrates. The relationships between the line width and scanning speed at various pulse energies on glass and PDMS substrates are shown in Figure 4a,b, respectively. All line widths on the PDMS substrates were larger than those on the glass substrates. These results suggest that the low thermal conductivity of the PDMS induced heat accumulation and thermal dispersion, indicating that the Cu precipitated area was expanded around the laser-irradiated one. The line width is expected to be reduced by decreasing the repetition frequency of the femtosecond laser pulses to inhibit heat accumulation.

### 3.2. XRD Spectra of the Patterns on Glass and PDMS Substrates

**Figure 5:**
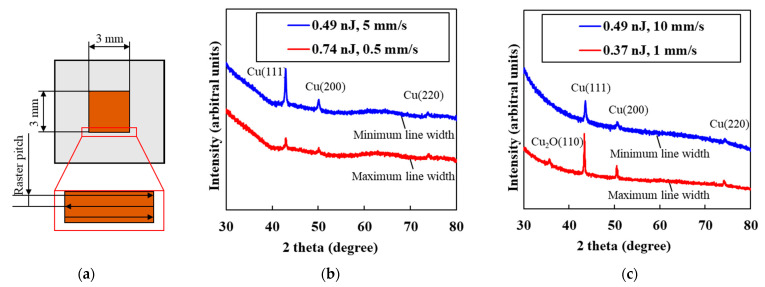
(**a**) Schematic illustration of the patterns fabricated by raster scanning of the focused femtosecond laser pulses. (**b**,**c**) XRD spectra of the patterns fabricated under the conditions that maximum and minimum line width were formed on glass and PDMS substrates, respectively.

The crystal structures of the patterns with the size of 3 × 3 mm were examined. The patterns were fabricated by raster scanning using the conditions that the maximum and minimum line width were formed on glass and PDMS substrates, respectively. Figure 4a illustrates the schematic illustration of the patterning method for the evaluation. The patterns were fabricated by raster scanning of the focused femtosecond laser pulses. The raster pitch was decided by considering the line width, the minimum and maximum line width of 10.8 and 28.1 µm, respectively, on glass substrates, and 11.1 and 52.7 µm, respectively, on PDMS substrates. Figure 5b,c show the XRD spectra of the patterns fabricated on glass and PDMS substrates, respectively. Only the pattern on PDMS substrates fabricated using the condition for maximum line width exhibited peaks corresponding to Cu_2_O, although there were only the diffraction peaks corresponding to Cu in all the other patterns fabricated on glass and on PDMS substrates. These results indicate that overheating of the patterns induced reoxidation in air. The minimum resistivity of the patterns on PDMS substrates is 1.4 × 10^−5^ Ωm, which is 10 times higher than that on glass substrates, indicating that microcracks formed by thermal shrinkage of the substrates during the laser irradiation increase the resistivity. The surface of the PDMS substrates was partially damaged by heat accumulation at low scanning speed in Figure 3. The cracks of Cu seemed to be induced by the thermal shrinkage of the PDMS substrate during the irradiation. Therefore, the electrical resistivity significantly decreased at the scanning speed of 1 mm/s and pulse energy of 0.37 nJ.

### 3.3. Resistivity of the Patterns on Glass and PDMS Substrates

**Figure 6:**
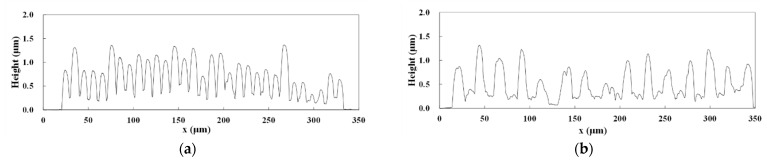

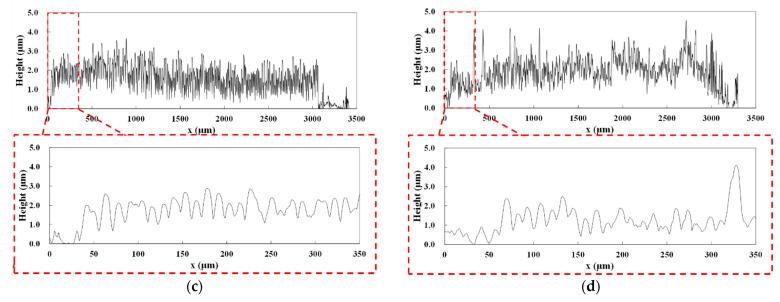
Cross-sections of the patterns. (**a**) Patterns on glass substrates at a scanning speed of 5 mm/s and pulse energy of 0.49 nJ. (**b**) Patterns on glass substrates at a scanning speed of 0.5 mm/s and pulse energy of 0.74 nJ. (**c**) Patterns on PDMS substrates at a scanning speed of 10 mm/s and pulse energy of 0.49 nJ. (**d**) Patterns on PDMS substrates at a scanning speed of 1 mm/s and pulse energy of 0.37 nJ.

The resistivity of the patterns fabricated using the conditions that the maximum and minimum line widths were formed on glass and PDMS substrates. The cross-sections of the patterns were evaluated using a surface profilometer. Figure 6 shows the cross-sections in the vertical direction of the raster scan. The periods of the pattern surfaces were the same as the respective raster pitches. The height of the patterns at the center of the focal spot was higher than that at far from the focal spot, indicating that the mass of the Cu precipitation increased with the intensity of the laser pulses. Table 1 shows the resistivities calculated using the resistance, cross-section, and length of the patterns. The minimum resistivity of the patterns on glass substrates and PDMS substrates were 7.3 × 10^−6^ Ωm and 1.4 × 10^−5^ Ωm, respectively. The resistivity of the patterns on glass substrates was almost the same as that of the patterns fabricated using tightly focused femtosecond laser pulses [14]. 

### 3.4. Cu Patterning on PDMS Step Structures

**Figure 7:**
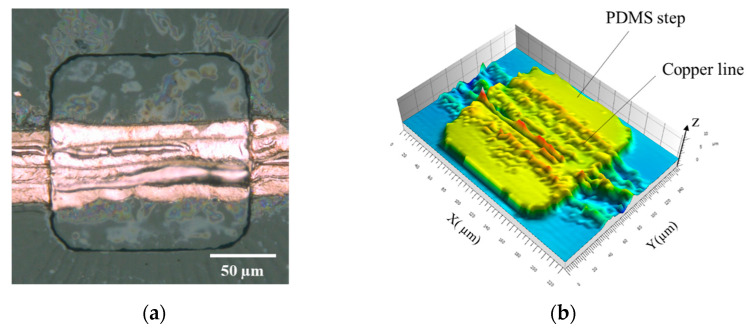
(**a**) Optical microscope image of the patterns on a PDMS step structure (top view). (**b**) 3D mapping of the fabricated Cu pattern.

The direct writing technique on PDMS substrates allows fabricating Cu patterns on nonplanar surfaces. We demonstrated the fabrication of the Cu pattern on the PDMS step structure (Figure 7). The height and the size of the step are 4 µm and 0.15 mm × 0.15 mm, respectively. By considering to the refractive index of the GACu complex, approximately 1.5, the Rayleigh length was estimated to be 5.8 µm. Therefore, the laser pulses were focused onto the substrate surfaces, and the position was not changed even on the step surface because the height was lower than the Rayleigh length. The electrically conducive patterns were formed, indicating that the effects of the reflection and the shadows on the sidewalls were not so serious because PDMS exhibited a small difference of the refractive index between the GACu complex and high transparency at the wavelength of the femtosecond laser pulses. The resistance of the pattern is 3.5 × 10^−4^ Ωm, which is 10 times higher than that on PDMS planar substrates (Table 1). The result was thought to be that the line pattern on the edges of the PDMS step must be thinner than that on the planar substrates. The patterns with high electrical conductivity are expected to be formed by increasing the laser pulse energy and/or decreasing the scanning speed only at the edges.

## 4. Conclusions

In this study, we investigate the direct writing properties of Cu patterns using femtosecond laser pulse-induced thermochemical reduction. The films of GACu complex coated on glass and PDMS substrates were irradiated by focused femtosecond laser pulses using a low NA objective lens.

1. The width of the line patterns formed on PDMS substrates was larger than that on glass substrates under the same conditions, such as laser scanning speed and pulse energy, which is attributable to heat accumulation on PDMS substrates induced by its lower thermal conductivity. The line width is expected to be reduced by decreasing the repetition frequency of the femtosecond laser pulses to inhibit heat accumulation.
2. XRD peaks of the patterns fabricated on glass substrates corresponded to Cu without significant oxidation. By contrast, although Cu patterns were also formed on PDMS substrates at a scanning speed of 10 mm/s and pulse energy of 0.49 nJ, Cu_2_O was also generated under the overheating conditions at a scanning speed of 1 mm/s and pulse energy of 0.37 nJ.
3. All the patterns exhibited electrical conductivity on glass and PDMS substrates. The minimum resistivity of the patterns on PDMS substrates is 1.4 × 10^−5^ Ωm, which is 10 times higher than that on glass substrates, indicating that microcracks formed by thermal shrinkage of the substrates during laser irradiation increase the resistivity.

This direct writing technique on soft materials and on nonplanar surfaces is useful for fabricating flexible microdevices. We will investigate the resistivity changes by bending the substrates in the future for the application.

## Figures and Tables

**Table 1:** Resistivity of the patterns fabricated on glass and PDMS substrates under various laser irradiation conditions.

Irradiation Conditions	Resistivity (Ωm)	Resistance (Ω)	Cross-Section (µm^2^)	Length (mm)	Average Thickness (µm)
Patterns on glass substrates at a scanning speed of 5 mm/s and pulse energy of 0.49 nJ.	7.3 × 10^−6^	9.4	195	0.25	0.65
Patterns on glass substrates at a scanning speed of 0.5 mm/s and pulse energy of 0.74 nJ.	1.4 × 10^−2^	4.5 × 10^4^	163	0.25	0.54
Patterns on PDMS substrates at a scanning speed of 10 mm/s and pulse energy of 0.49 nJ.	1.4 × 10^−5^	16	4359	5.0	1.5
Patterns on PDMS substrates at a scanning speed of 1 mm/s and pulse energy of 0.37 nJ.	6.2	6.3 × 10^6^	4950	5.0	1.7
